# Kinematic effects of unilateral TKA on the contralateral knee in Chinese patients with advanced osteoarthritis: a prospective gait analysis study

**DOI:** 10.3389/fbioe.2024.1463049

**Published:** 2024-09-11

**Authors:** Haibo Wang, Wenhao Duan, Xiaodong Dang, Zhenxian Chen, Yinghu Peng, Shuxin Yao, Weijie Zhang, Jianbing Ma

**Affiliations:** ^1^ Graduate School, Xi’an Medical University, Xi’an, China; ^2^ Department of Joint Surgery, Honghui Hospital, Health Science Center, Xi’an Jiaotong University, Xi’an, China; ^3^ School of Construction Machinery, Chang’an University, Xi’an, China; ^4^ Research Center for Neural Engineering, Shenzhen Institute of Advanced Technology, Chinese Academy of Sciences, Shenzhen, China

**Keywords:** unilateral total knee arthroplasty, contralateral knee, knee osteoarthritis, knee kinematics, gait analysis

## Abstract

**Background:**

Patients with knee osteoarthritis (OA) who receive unilateral total knee arthroplasty (TKA) often report reduced pain and enhanced function in the untreated knee, yet the kinematic mechanisms are not fully understood. Our study aimed to clarify these effects through a gait analysis of the untreated knee following unilateral TKA.

**Methods:**

This study enrolled 118 end-stage OA patients with varus deformity scheduled for TKA, categorized into the contralateral osteoarthritis group (Contra-OA), consisting of patients with end-stage OA in both knees requiring surgical treatment, and the contralateral TKA group (Contra-TKA), which included patients who had undergone TKA on one knee and had end-stage OA in the untreated knee awaiting surgery. Kinematic data of the knee joint during treadmill walking were collected using the Opti_Knee gait analysis system, and a comparative analysis was conducted.

**Results:**

The Contra-TKA group exhibited improvements in step length, anterior-posterior translation, range of motion, vertical translation, and internal-external rotation compared to the Contra-OA group (*p*-values ranging from 0.0013 to 0.0463). Notable differences in flexion-extension angles and abduction/adduction rotation were also observed (*p* = 0.0013 and 0.0166, respectively). At the initial contact (IC), obvious differences in internal-external rotation, anterior/posterior translation, and vertical translation were noted. At the opposite toe-off (OT), significant differences in internal-external rotation. At the tibia vertical (TV) moment, significant differences were observed in all three translation indicators of joint translation. At other pivotal gait cycle points, vertical and anterior/posterior translations in Contra-TKA group continued to exhibit more meaningful decrease. Collectively, these findings underscore the protective kinematic effects of TKA on the untreated contralateral knee, indicating an improved biomechanical adaptation following TKA surgery.

**Conclusion:**

In summary, the study’s findings indicate that unilateral TKA imparts kinetic effects on the untreated contralateral knee, as evidenced by significant improvements in key gait parameters. These enhancements, observed at both initial contacts and throughout the gait cycle, suggest a positive biomechanical support post-TKA, might contribute to better gait efficiency and reduced load on the contralateral untreated knee.

## 1 Introduction

With the increase in aging population and the rise of risk factors such as obesity, the incidence of knee OA has risen significantly (Hao et al., 2024). Severe Knee OA could affect the quality of life of elderly patients, bringing a heavy economic and social burden (Perruccio et al., 2024). TKA is one of the most successful treatments for end-stage knee osteoarthritis, significantly reducing pain and improving postoperative knee joint function, including walking speed and endurance (Dávila Castrodad et al., 2019; Kolasinski et al., 2020; Konnyu et al., 2023). Patients with knee OA who undergo TKA often show degeneration in both knees (Piovan et al., 2022). However, the debate over whether to perform simultaneous bilateral TKA or to conduct TKA surgery in stages continues (Parisi et al., 2018b; Smith et al., 2013; Stefánsdóttir et al., 2008; Tanpure et al., 2023). Research by Stefánsdóttir and colleagues (Stefánsdóttir et al., 2008) in Sweden showed that the 30 day mortality risk of simultaneous bilateral surgery is 1.94 times of that of staged bilateral knee replacement surgery, making unilateral TKA safer, even for physically healthy patients. Clinically, mainstream medical centers predominantly emphasize unilateral TKA.

It is often reported that patients who have undergone unilateral TKA experience some extent of improvement in pain and function of the contralateral knee with osteoarthritis, an effect known as the “splinting effect” (Fleming et al., 2021). Parisi and colleagues (Parisi et al., 2018a) analyzed the full-length radiographs of the lower limbs of 203 patients who underwent unilateral TKA and found that 6 weeks after the initial unilateral TKA, the mechanical axis of the contralateral lower limb improved by about 1°, with more improvements in patients with preoperative varus deformity of the contralateral knee. Smith et al. (2013) found that after unilateral TKA, the patient-reported outcome measure scores and knee function of both the operated and contralateral knees improved over time. Tanpure et al. (2023) plantar pressure research found that after unilateral TKA, the contralateral lower limb showed improvements in joint angle, knee flexion-extension, ankle dorsiflexion and plantarflexion, and pelvic tilt at standing phase, which helps doctors guide patients to adjust the step of the contralateral limb after TKA to delay the progression of osteoarthritis. However, the mechanism behind the improvement in symptoms of contralateral knee OA after TKA is still unclear.

The evaluation of surgical outcomes for knee OA mainly includes three methods: physical function, clinical scales, and gait assessments (Choi et al., 2021). Imaging can reflect the structural condition of patients’ joints but is difficult to obtain their locomotion function. Clinical scale assessments often rely on doctors’ clinical experience, which is highly subjective and variable. Compared to the first two types of assessments, gait assessment is more objective, stable, and highly repeatable (Zeng et al., 2022a), and the kinematic indicators of the knee joint during the gait cycle are of great significance for the diagnosis, treatment, and efficacy evaluation of knee joint diseases (Kwon et al., 2020; Li et al., 2022). Gait assessment provides useful ways to reveal the mechanism behind the improvement in symptoms of contralateral knee OA after TKA.

Therefore, this study attempts to apply a valid optical knee analysis system to collect preoperative six degrees of freedom (6-DOF) knee joint kinematics of patients with knee OA awaiting TKA surgery and using patients with bilateral knee OA as a control, aiming to analyze the changes in kinematic parameters of the contralateral knee after TKA from a kinematic perspective, and to clarify the potential impact of unilateral TKA on the patient’s contralateral OA knee kinematics.

## 2 Methods

### 2.1 Participants

The study received ethical approval from the Xi’an Honghui Hospital of Xi’an Jiaotong University (Approval No. 202407002). We enrolled 118 patients with knee OA and varus deformity, scheduled for surgery at the hospital’s Joint Department from November 2023 to May 2024. Eligibility criteria included: diagnosis of knee OA, age between 40 and 80 years old, presence of varus deformity (all varus deformity meet the need of gait gathering), a minimum 6-month interval since the last unilateral TKA and the requirement for surgical intervention on both knees as determined by standard clinical practice and assessment. Exclusions applied to patients with a history of joint trauma or surgery, inflammatory arthritis, neurological disorders affecting muscle strength, inability to cooperate with gait assessment, history of fragility fractures or falls, patients with unilateral OA where the contralateral knee does not require surgical intervention, and patients with a valgus knee deformity, as well as any factor might impede gait data collection.

We documented demographic data including gender, age, height, weight, and body mass index. Of the 118 patients, 89 had bilateral knee osteoarthritis without TKA, while 29 had unilateral TKA with no surgery on the contralateral knee for at least 6 months post-surgery, as TKA patients typically reach a stable and satisfied walking speed and overall knee function by 6 months post-surgery (Kennedy et al., 2008; Ekhtiari et al., 2024).

Participants were categorized into two groups based on the condition of their opposite knee: the Contra-OA group comprised patients with untreated bilateral OA, and the Contra-TKA group included those who had not received a second TKA at least 6 months after the initial procedure.

In this research, all gait data were obtained preoperatively. Patients with bilateral knee osteoarthritis in the Contra-OA group provided pre-TKA gait data for both knees, with one set selected at random, yielding 89 valid datasets. For the Contra-TKA group, we obtained gait data from the untreated knee before the second TKA, mirroring the initial collection process, providing 29 valid datasets. Baseline characteristics are detailed in Table 1.

**TABLE 1:** Description of demographic characteristics of Contra-OA group and Contra- TKA group (mean ± SD).

	Contra-OA group	Contra-TKA group	*p*-value
Sex, n (%)			0.272
Female	62 (69.66)	17 (58.62)	
Male	27 (30.34)	12 (41.38)	
Age (year)	66.00 ± 7.89	64.97 ± 6.18	0.521
Height (cm)	161.42 ± 7.47	162.34 ± 7.82	0.566
Weight (Kg)	67.54 ± 11.29	64.86 ± 10.91	0.268
BMI (kg/m^2^)	25.90 ± 3.89	24.68 ± 3.81	0.144

Abbreviations: SD, Standard Deviation; BMI, Body Mass Index.

### 2.2 Experimental equipment

**FIGURE 1:**
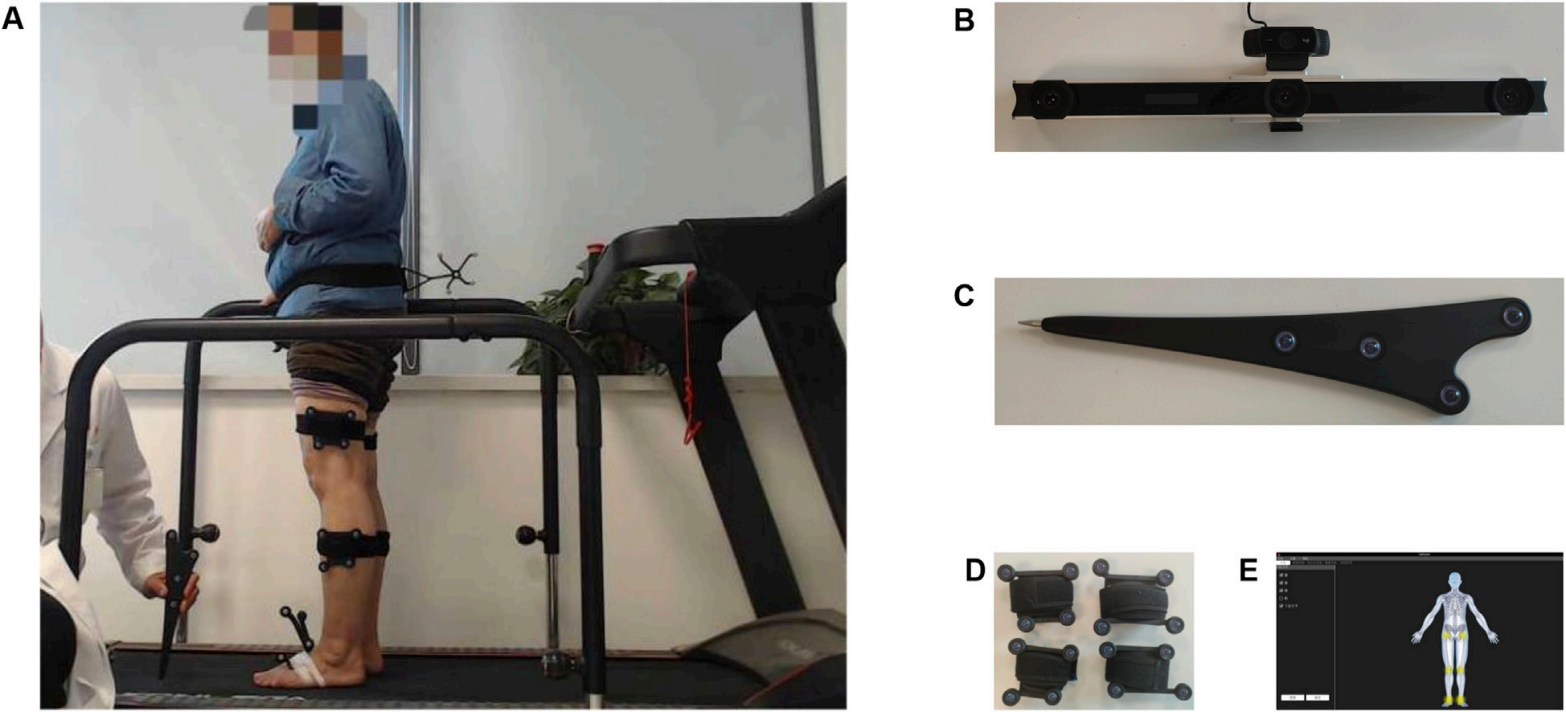
**(A)** Anatomical landmark localization. **(B)** Surgical navigation stereo-infrared tracking device and High-speed optical camera, **(C)** Handheld probe with infrared markers, **(D)** Infrared light-reflecting Markers, **(E)** Workstation computer.

We utilized an optical marker-based gait analysis system (Opti_Knee, Innomotion, Inc, Shanghai, China) to collect the knee joint motion data of the subjects (Zhang, Yao, Wang, Huang, et al., 2015). The accuracy of this system has been validated through research (Fung et al., 2018; Wang et al., 2021). The setup included a workstation with custom software for immediate motion analysis (Figure 1E), a stereo infrared tracking system for surgical navigation (NDI Polaris Spectra, Northern Digital, Canada) (Figure 1B), two sets of infrared light-reflecting markers (OK_Marquer1, Innomotion Inc., Shanghai, China) (Figure 1D), a high-speed optical camera (Figure 1B), and a handheld digital probe (Figure 1C). The stereo infrared tracking system possesses a 60 Hz sampling rate and a 0.3 mm root mean square (RMS) accuracy for the tracking device (Elfring et al., 2010). The system guarantees rotational repeatability under 1.3° and translation repeatability under 0.9 mm (Zhang et al., 2016). The study was performed on a dual-belt treadmill with the system settings kept constant.

### 2.3 Experimental procedure

As Figure 1A showed, patients were prepared by exposing their lower limbs and assuming a standard standing posture with folded arms to prevent marker obstruction. With the patient in a standing position, we affixed reflective markers capable of reflecting light to the patient’s thigh and shin. Subsequently, while one trained researcher operated the accompanying software on a computer, another researcher used an infrared reflective marker probe to sequentially identify seven bony landmarks on the patient’s anatomic site (including greater trochanter, lateral epicondyle, medial epicondyle, medial malleolus, lateral malleolus, and the second metatarsal). After the identification process, the system’s software automatically established personalized three-dimensional coordinate systems for the femur and tibia., referencing the geometric relationships of these landmarks to the hip or tibial rigid plate.

During the treadmill walking phase (treadmill slope was set to 0° constantly), under safe conditions, patients were guided by trained staff to walk at their comfortable pace, which was gradually adjusted and recorded. Post-adaptation, kinematic data were collected over a 15-second interval of barefoot walking, with repetitions to secure a minimum of four valid data sets. During our data collection, each patient’s gait data were recorded independently for both legs in the contra-OA group. The process was as follows: the patient walked at a comfortable pace with the right leg aligned towards the tracking device. After the right leg data collection, the patient rested. Then, the patient stepped back and the treadmill belt direction was set reversely to face the left leg towards the tracking device, mirroring the initial setup for the right leg. One set was selected at random in the Contra-OA group for each patient.

During gait data collection and analysis, unique challenges can occur, like elderly patients experiencing knee pain that prevents full participation or those too anxious to walk on a treadmill as they normally would. To maintain data integrity, researchers often exclude such patients from the final statistical analysis, regardless of whether they formally meet exclusion criteria.

The gait system’s software (Optimum, Innomotion, Inc., Shanghai, China) was applied to process the collected data, automatically identifying averages and exporting key metrics such as the maximum, minimum, and range of motion for the knee joint’s 6-DOF across a gait cycle, along with step length and cadence. The 6-DOF of the knee joint kinematic indicators include flexion/extension, external/internal rotation, adduction/abduction, posterior/anterior translation, proximal/distal translation, and medial/lateral translation (Zhang et al., 2015). This helps clinical doctors to dynamically evaluate the knee joint function of KOA patients (Zeng et al., 2022a). By exploring the changes in knee kinematics, an overall view of the factors affecting the knee joint can be obtained (Yang et al., 2022).

A complete gait cycle is divided into the stance and swing phases, encompassing seven pivotal events: four during the stance phase—initial contact (IC), heel rise (HR), opposite toe-off (OT), and opposite initial contact (OI)—and three during the swing phase: feet adjacent (FA), tibia vertical (TV), and toe-off (TO) (Constantinou et al., 2017), To analyze the kinematic changes at critical junctures of the gait cycle, we focus on seven key points: IC at approximately 0% of the gait cycle, OT at about 12%, HR at around 32%, OI at roughly 52%, TO at about 62%, FA at approximately 76%, and TV at roughly 85% (Narayanan, 2007; Zeng et al., 2022a). This approach allows for a detailed examination of the dynamics at each stage of the gait.

### 2.4 Data processing

The gait system’s companion software determined the spatial coordinates of anatomical landmarks, such as the femur and tibia, calibrated earlier. It utilized the geometric relationships between these landmarks and either the hip joint or the tibial rigid plate at each frame of the gait cycle to establish a 3D coordinate system for both the femur and tibia. The relative translation between the tibial and femoral coordinate systems quantified the 3D motion of these bones (Dyrby and Andriacchi, 2004). The tibia’s rotation relative to the femur was calculated using a sequence of Euler angles: medial-lateral, anterior-posterior, and proximal-distal (Zhang et al., 2015).

The Optimum software was then employed to compute the knee joint’s six degrees of freedom (6-DOF)(Chao et al., 1983). By dividing each gait cycle into 100 equidistant points, an average cycle was derived (Gao and Zheng, 2010; Li et al., 1996). This method allowed for the calculation and comparison of cascade, step length, and the knee joint’s 6-DOF maximum, minimum, and range of motion.

### 2.5 Statistical analysis

Data were processed and statistically analyzed using SPSS v26 software (SPSS Inc., IL, United States). Participants were categorized into two groups according to the status of their contralateral limb: the OA group and TKA group, known as the Contra-OA and Contra-TKA groups, respectively. Normality of the data was evaluated with the Shapiro-Wilk test. Metric data conforming to a normal distribution were compared between groups using the *t*-test, while non-normally distributed data were analyzed with the Mann-Whitney U test. A *p*-value ≤0.05 was set as the threshold for statistical significance, with results presented as Mean ± SD. For graphical representation, GraphPad Prism version 9.4.1 (GraphPad Software, Inc., San Diego, CA) and R-4.3.3 software (R Foundation for Statistical Computing, Vienna, Austria) were utilized.

## 3 Results

### 3.1 Patients’ demographic data

The mean age of patients in the Contra-OA group was 66.00 ± 7.89 years, while the Contra-TKA group had a mean age of 64.97 ± 6.18 years. Gender distribution did not differ significantly between the groups (*p* = 0.272). Demographic characteristics, detailed in Table 1, revealed no statistically significant difference in body mass index (BMI), weight, height, or age among the groups (*p* > 0.05).

### 3.2 Step length, gait velocity and cadence analysis

**FIGURE 2:**
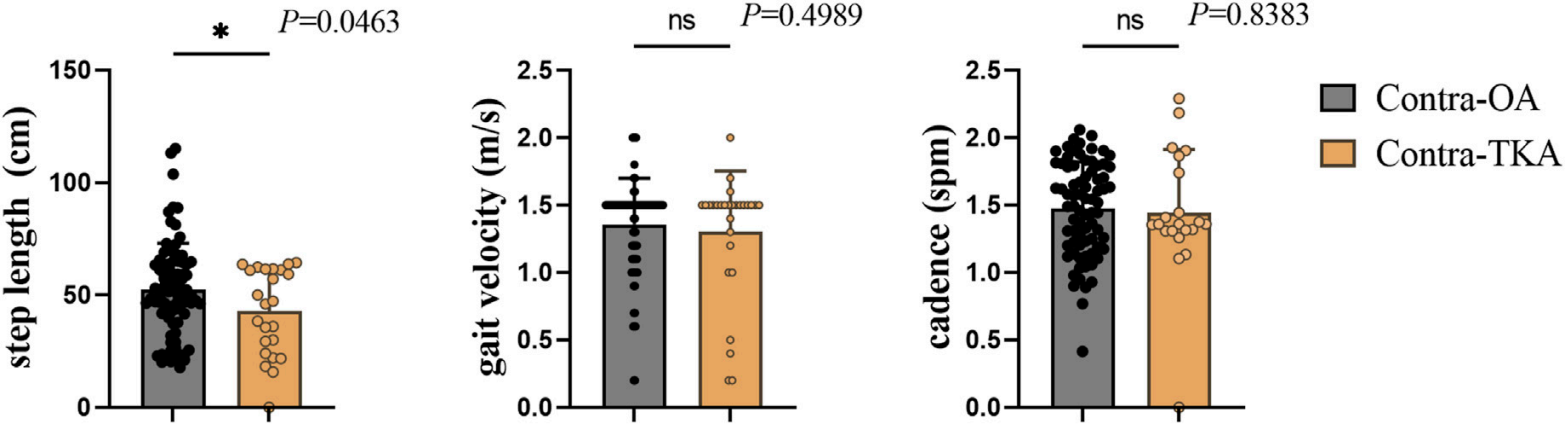
The comparison between the Contra-OA group and the Contra-TKA group in terms of step length, gait velocity and stride frequency, * The significant difference is set at the 0.05 level.

This study analyzed gait parameters in 118 osteoarthritis knee joints, with 89 in the Contra-OA group and 29 in the Contra-TKA group. Figure 2 displays step length, gait velocity, and cadence for both groups. There is a significant difference in step length (*p* = 0.0463), while there is no significant difference in gait velocity and cadence. This lack of difference may be due to the treadmill’s specific gait collection conditions.

### 3.3 Analysis of rotational parameters

**FIGURE 3:**
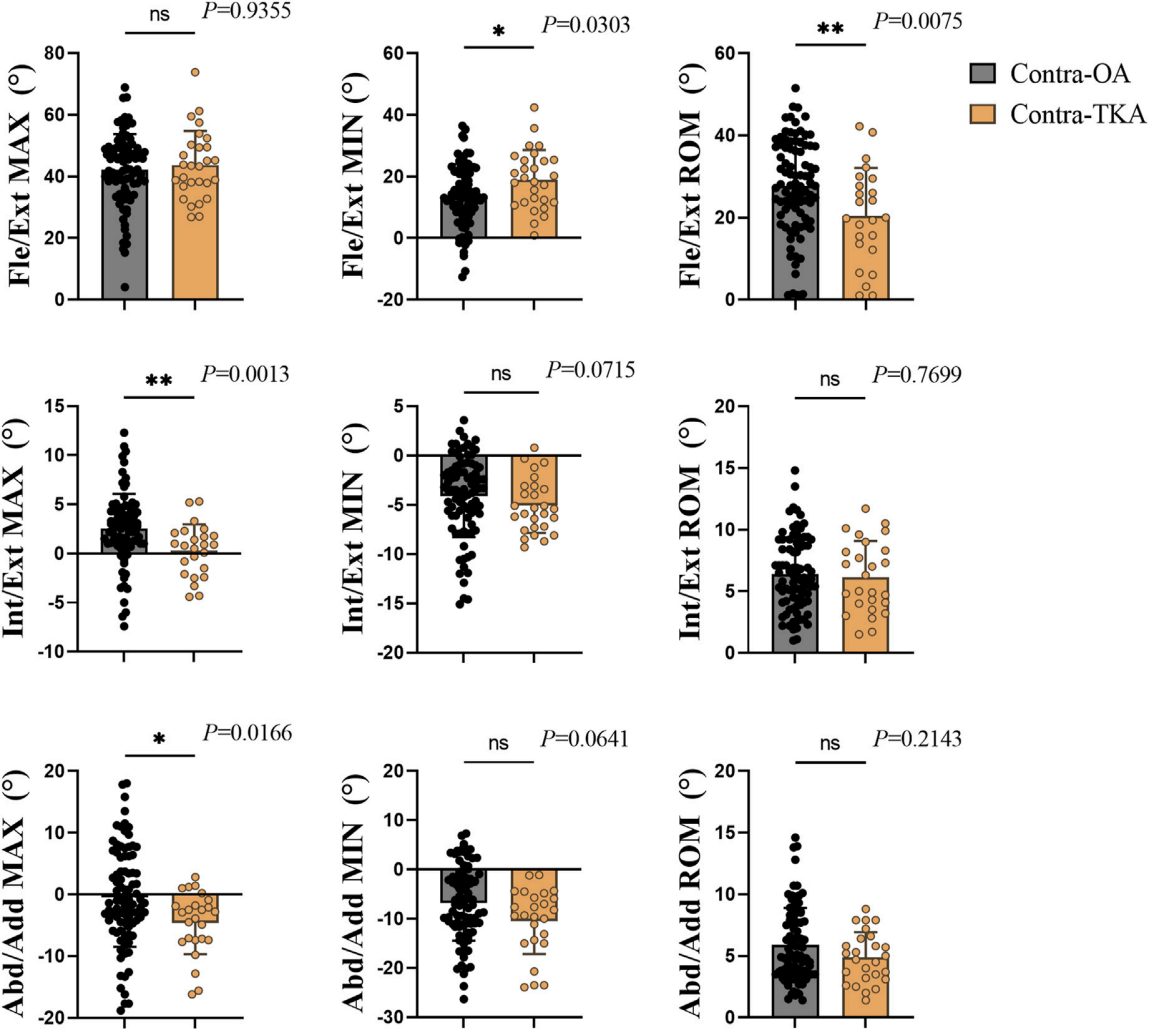
Rotation indicators of the knee joint, with each column from left to right representing the maximum value (Max), minimum value (Min), and range of motion (Rom) for each respective direction of movement. For flexion, external rotation, and abduction, the maximum and minimum values are marked as positive (+); for extension, internal rotation, and adduction, they are marked as negative (−). ROM values are all positive. Each row from top to bottom represents Flex/Ext, Flexion/Extension; Int/Ext, Internal/External Rotation; Abd/Add, Abduction/Adduction. * The significant difference is set at the 0.05 level.

Figure 3 illustrated the differences in the maximum, minimum, and range of motion for key rotational parameters of the knee joint—specifically, the flexion/extension, internal/external rotation, and abduction/adduction angles—between the Contra-OA and Contra-TKA groups. The analysis revealed significant differences in the minimum flexion/extension angle and its range of motion, with the Contra-TKA group exhibiting an increased minimum angle and a reduced range (*p* = 0.0303 and *p* = 0.0075, respectively). The maximum internal/external rotation angle also showed a significant reduction in the Contra-TKA group, whereas the maximum abduction/adduction rotation angle was notably higher (*p* = 0.0013 and *p* = 0.0166, respectively). These results suggest a substantial impact of TKA on the rotational dynamics of the contralateral knee, indicative of a protective effect on joint kinematics.

### 3.4 Analysis of translation parameters

**FIGURE 4:**
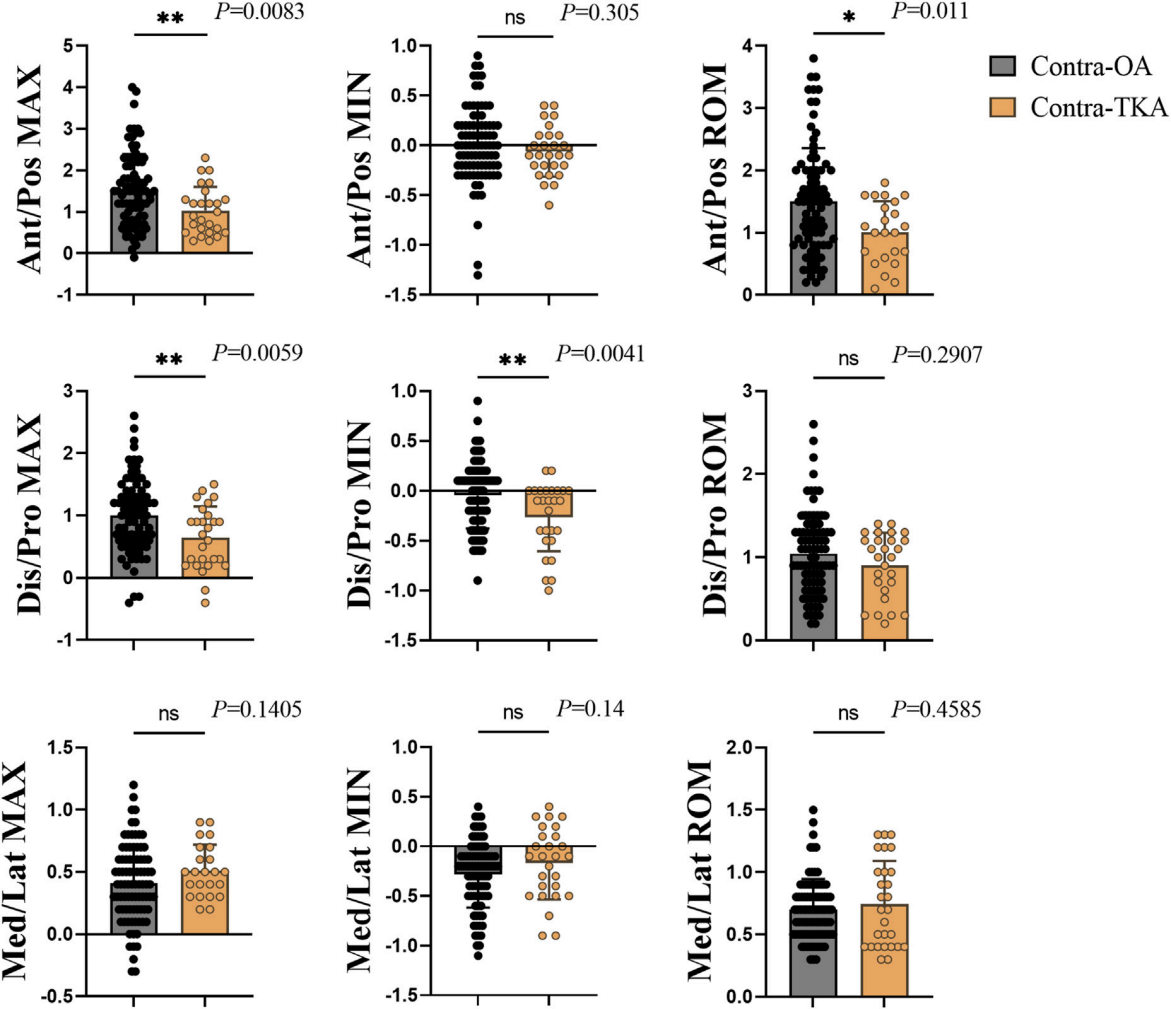
Translation indicators of the knee joint, with each column from left to right representing the maximum value (Max), minimum value (Min), and range of motion (Rom) for each respective direction of movement. For anterior translation, proximal translation, and lateral translation, the maximum and minimum values are marked as positive (+); for posterior translation, distal translation, and medial translation, they are marked as negative (−). ROM values are all positive. Each row from top to bottom represents Ant/Pos, Anterior/Posterior translation; Dis/Pro, Distal/Proximal translation; Med/Lat, Medial/Lateral translation. * The significant difference is set at the 0.05 level.

Figure 4 illustrated the translation movement of the knee joint, with significant variations noted in anterior/posterior translation between the Contra-OA and Contra-TKA groups. The maximum translation and range of motion were considerably lower in the Contra-TKA group, with respective *p*-values of 0.0083 and 0.011, indicating a reduced extent of anterior/posterior movement post-TKA. For the distal/proximal translation, the study identified significant differences in both maximum and minimum values, with *p*-values of 0.0059 and 0.0041. The Contra-TKA group showed a lower maximum translation, and a higher minimum translation compared to the Contra-OA group, reflecting a change in the vertical translation pattern following surgery. In contrast, the medial/lateral translation parameters did not exhibit significant differences between the Contra-OA and Contra-TKA groups, indicating that TKA does not significantly influence the mediolateral shift of the contralateral untreated knee to a notable extent.

### 3.5 Gait cycle kinematic analysis

**FIGURE 5:**
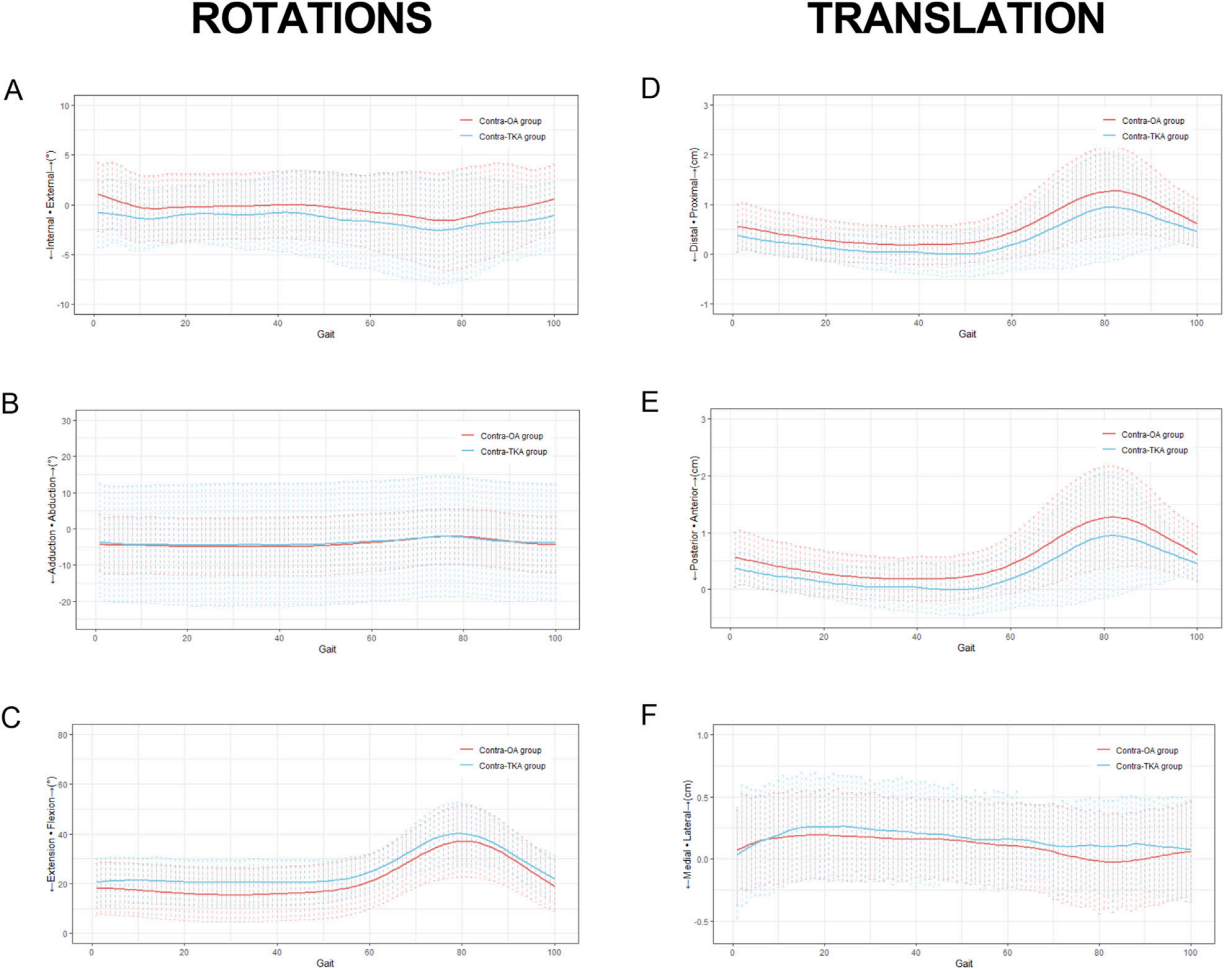
Kinematic variations of the knee joint measured during treadmill gait. The thick solid line represented the average movement of the femur relative to the tibia for 663 the subjects, and the range of the dashed line represents the standard deviation. The patterns started with heel strike and ends with the heel strike of the contralateral foot from left to right, the first column showed the changes of different rotation indicators during the entire gait cycle, labeled as **(A)** Internal/External; **(B)** Adduction/Abduction. **(C)** Extension/Flexion. The second column showed the changes of different translation indicators during an entire gait cycle, labeled as **(D)** Distal/Proximal translation; **(E)** Posterior/Anterior translation; **(F)** Medial/Lateral translation.

The kinematic variations of the knee joint during the entire gait cycle for the Contra-OA group and the Contra-TKA group were depicted in Figure 5. As shown in Figure 5A, the Contra-TKA group showed a reduced external rotation angle in the late swing and early stance phases compared to the Contra-OA group. Figure 5C demonstrated that the Contra-TKA group consistently presented with a higher flexion angle across the entire gait cycle. Figure 5D indicated lesser Distal/Proximal translation in the Contra-TKA group throughout the gait cycle. Additionally, Figure 5E revealed that during the swing phase, the Contra-TKA group experienced less anterior-posterior translation than the Contra-OA group. In contrast, Figures 5B, F showed no significant differences in abduction/adduction rotation angle and medial/lateral translation between the two groups, implying that TKA does not markedly affect these kinematic aspects of the contralateral knee joint. This analysis of the gait cycle kinematics provides a detailed perspective on how TKA influences the dynamic movement patterns of the contralateral knee, highlighting both the changes induced by the TKA surgery and the elements that remain unaffected.

### 3.6 Kinematic variations at gait cycle key events

**FIGURE 6:**
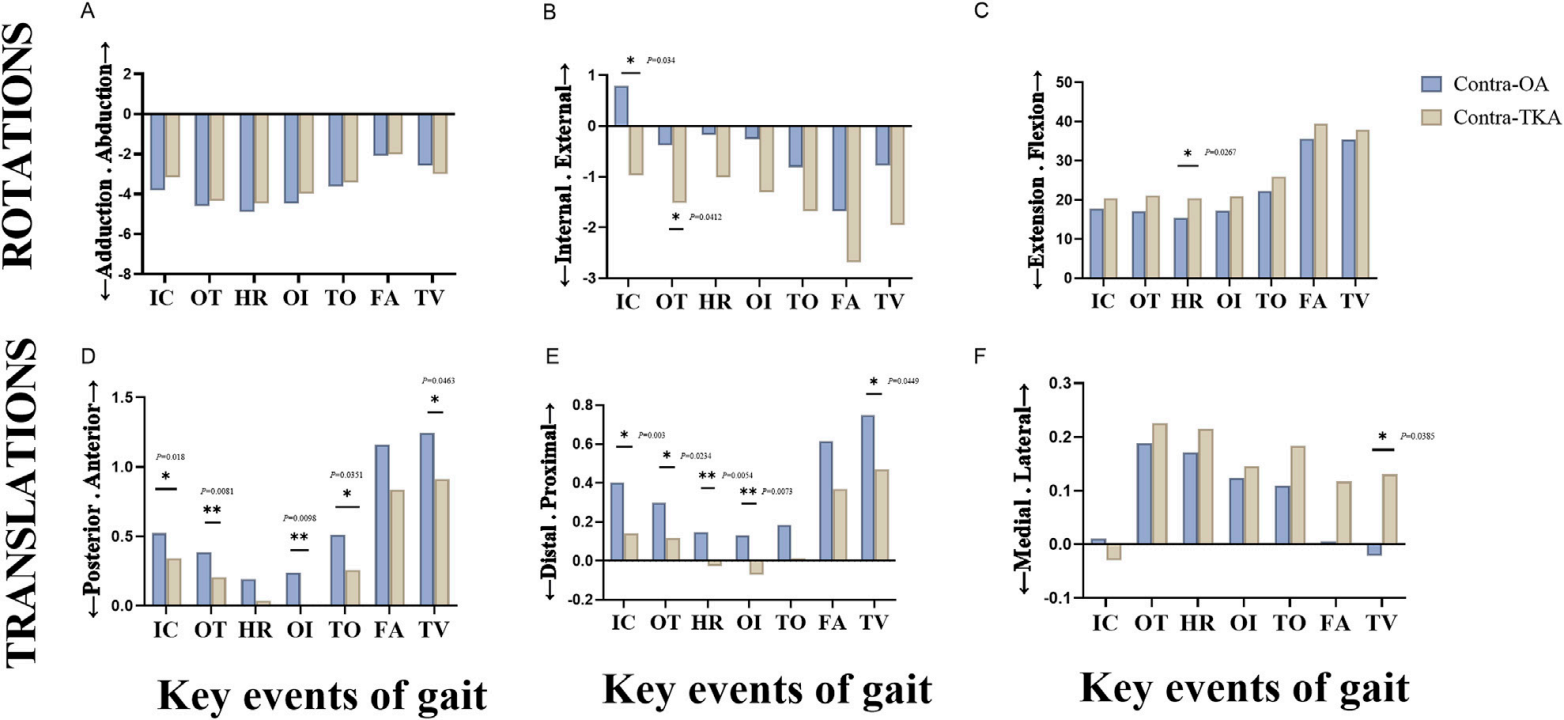
A kinematic comparison of the knee joint between the Contra-OA group and the Contra-TKA group at seven key events during the entire gait cycle. The first row showed the comparison of the three rotational indicators of the knee joint, labeled as **(A)** Adduction/Abduction; **(B)** Internal/External; **(C)** Extension/Flexion. The second row showed the comparison of the three translational indicators of the knee joint, labeled as **(D)** Posterior/Anterior translation; **(E)** Distal/Proximal translation; **(F)** Medial/Lateral translation. The key events include initial contact (IC), opposite toe-off (OT), heel rise (HR), opposite initial contact (IO), toe-off (TO), feet adjacent (FA), and tibia vertical (TV). * The significant difference is set at the 0.05 level.

Figure 6 presented the kinematic changes of the knee joint at seven pivotal moments within the gait cycle for both the Contra-OA and Contra-TKA groups. As shown in Figure 6A, the Contra-TKA group displayed a trend toward lower adduction/abduction rotation angles at six events excepting TV, though the differences were not statistically significant. Figure 6B showed that at the IC, the Contra-TKA group exhibited a restoration of normal tibial internal rotation, contrasting with the abnormal external rotation observed in the Contra-OA group. At the OT, the external rotation in the Contra-TKA group significantly improved. Figure 6C indicated that the Contra-TKA group had increased flexion angles at all seven key events, with a significant increase at the HR event. Figure 6D indicated that the knee joint’s anterior/posterior movement in the Contra-TKA group was significantly lower than in the Contra-OA group, except at HR and FA moments.

Figure 6E revealed that, except at the TO event and FA event, the Contra-TKA group experienced significantly less distal translation at five key time points compared to the Contra-OA group. Figure 6F illustrated that at TV, the tibia in the Contra-TKA group moved significantly laterally, while in the Contra-OA group, it moved medially. These results offer insights into TKA’s effects on knee joint kinematics during key gait phases, especially partial restoration of normal movement and a reduction in translational movements at specific events.

## 4 Discussion

TKA has been proven to be one of the most successful treatments for end-stage knee osteoarthritis, significantly reducing pain and improving postoperative knee joint function (Dyer, 2012; Kolasinski et al., 2020; Konnyu et al., 2023; Spieth et al., 2016). In this study, the sophisticated optical knee analysis system was adopted to collect preoperative 6-DOF gait data from patients with knee osteoarthritis awaiting TKA surgery and used patients with bilateral knee osteoarthritis as a control group, aiming to elucidate the impact of unilateral TKA on the kinetics of the contralateral knee joint in patients.

The research revealed that patients with Contra-TKA exhibited reduced step length, diminished maximum anterior/posterior and Distal/Proximal translations, internal/external and Abduction/Adduction rotations, decreased minimum Distal/Proximal translation, decreased range of motion in knee flexion/extension rotation and Anterior/Posterior translation, compared to those with Contra-OA. Conversely, the Contra-TKA group had a higher minimum in flexion/extension rotation. The underlying mechanism for this phenomenon might be attributed to the reduced load on the TKA group’s opposite knee.

Previous studies have consistently highlighted the beneficial impact of unilateral TKA on the contralateral knee affected by OA.

Parisi’s study (Parisi et al., 2018a) noted that unilateral TKA in patients with bilateral OA led to improved lower limb alignment, particularly in those with significant varus deformities, potentially delaying the need for TKA on the opposite knee. Smith et al. (2013) reported significant improvements in the WOMAC scores of the non-operated knee in such patients. Tanpure et al. (2023)’s research observed enhancements in stance phase, joint angles, and movement patterns in the contralateral limb post-TKA, aiding in therapeutic gait adjustments to mitigate OA progression. Pfeufer et al. (2022) utilized wearable sensors to demonstrate reduced pain and increased load bearing in the operated knee, with corresponding improvements in gait. Henriksen et al. (2006) study corroborated these findings, showing that pain relief post-TKA enhanced load bearing on the operated side, alleviating stress on the contralateral knee and slowing OA progression. Based on these insights, this study further explored the impact of the operated knee joint on the non-operated knee joint after unilateral TKA from the perspective of gait analysis.

Step length, a pivotal metric in gait analysis, measures the distance from one heel-strike to the subsequent heel-strike on the foot within a walking stride (Stebbins et al., 2023). Knee OA patients frequently exhibit reduced step length on the affected side due to pain and stiffness (Choursiya et al., 2024; Park et al., 2021). In this study, the shorter step length observed in the Contra-TKA group could be attributed to patients’ protective responses towards their untreated knee. In patients with one knee replaced (Contra-TKA group), the treated side seemed to lead, prompting the untreated side to quickly follow, potentially driving the overall gait pattern. This dynamic suggests that the operated knee may influence the movement of the non-operated knee, affecting gait (Pfeufer et al., 2022). Thus, TKA on one side might offer protective benefits to the non-operated knee joint, we interpret this gait adaptation as a strategic response to pain and mobility constraints.

Gait speed is an essential measure in gait analysis, with slower speeds often linked to worse functional outcomes for knee osteoarthritis (KOA) patients, as noted in previous studies (Akkan et al., 2024; Kroman et al., 2014; Lozano-Meca et al., 2024). In our study, gait data was collected using a treadmill. Patients, who were in severe pain and could not maintain a constant speed, were allowed to walk at a comfortable cadence after an adaptation period, ensuring an ethical and safe data collection method. Figure 2 showed no significant differences in gait velocity, and cadence between the groups, possibly due to the treadmill setup. The slightly slower speeds observed in the contra TKA group might be because of the increased reliance on the TKA side for support.

Regarding gait rotation indicators, the group with Contra-TKA demonstrated notable differences from the Contra-OA group across in some rotational parameters. The knee joint’s movement around the vertical axis in the horizontal plane, known as the internal/external rotation angle, is vital for knee joint flexibility and function (Zeng et al., 2022b). Our findings indicate an increased tibial internal rotation angle in the Contra-TKA group, particularly at the IC event, where a significant divergence between the groups was observed. The Contra-TKA group exhibited increased internal rotation, while the Contra-OA group showed external rotation (as shown in Figure 6B). Additionally, the maximum internal/external rotation angle of the knee was significantly diminished in the Contra-TKA group (as shown in Figure 3). This alteration could be attributed to the fact that the surgically recovered knee in the Contra-TKA group assumes a greater load-bearing role, serving as the primary weight-bearing limb. Consequently, the non-operated knee experiences reduced pain and displays a physiological internal rotation function at the IC moment (Khambete et al., 2021; Li et al., 2023; Roach et al., 2022). In contrast, the Contra-OA group exhibited abnormal tibial external rotation, highlighting the potential protective and supportive effect of surgical TKA on the non-operated knee in patients with knee OA.

In the coronal plane, the knee joint’s movement around its normal axis is characterized by the adduction/abduction angle, which is key to understanding knee joint dynamics (Zeng et al., 2022b). Our study noted a decrease in the knee joint’s adduction angle in the Contra-TKA group when compared to the Contra-OA group, with a significant reduction in the maximum adduction/abduction angle. The reduction in the knee joint’s adduction angle in the Contra-TKA group could be attributed to the TKA limb’s supportive role, which alleviates the weight-bearing burden on the non-operated knee. This support negates the need for a larger adduction/abduction angle to facilitate walking, thereby demonstrating the potential protective effect of TKA on the non-operated knee in OA patients.

In the sagittal plane, the knee joint’s flexion/extension angle is a critical measure of its mobility (Zeng et al., 2022b). This angle significantly influences daily activities like walking and stair climbing, and thus, the quality of life. Our study observed reduced Flexion/Extension ROM and higher flexion angle at HR for the Contra-TKA group. This alteration may be due to the TKA limb’s enhanced pain relief and increased load-bearing capacity post-surgery, which in turn lessens the load and functional demands on the non-operated knee, enhancing its function. Furthermore, the minimum flexion/extension angle in the Contra-TKA group was significantly higher than in the Contra-OA group, suggesting that patients have greater confidence in extending their knee to the optimal angle necessary for walking activities. This finding indicates that TKA not only alleviates pain but also potentially improves the overall gait and functional capacity of patients with knee OA.

Joint translation indicators are essential for assessing the functionality of the knee joint. Compared to the Contra-OA group, the Contra-TKA group showed decreased distal/proximal translations and anterior/posterior translations. Significantly, the tibial translation relative to the femur was notably reduced in the TKA group’s contralateral knee across most of key gait cycle events.

Distal/proximal translation, which refers to the vertical movement of the knee joint, is pivotal for movement function. It indicates the relative distance between the femur and tibia during the swing and support phases of walking, thus reflecting the knee joint’s load-bearing status (Zeng et al., 2022a). During normal walking, the distal/proximal translation of the knee joint is an indicator of the joint’s load-bearing status. It measures the vertical movement of the tibia in relation to the femur, which occurs during both the swing and stance phases of the gait cycle. This translation is a critical component of the knee’s biomechanics, as it directly correlates with the joint’s ability to distribute and manage the body’s weight effectively during movement. The reduction in distal/proximal translation in the Contra-TKA group is likely due to the TKA side’s pain relief, which allows it to bear more weight. This shift in load bearing might reduce the load on the non-operated knee, resulting in a lower distal/proximal translation. This change underscores the TKA’s protective effect on the non-operated knee by reducing its load bearing demands and potentially enhancing its function.

The anterior/posterior translation of the knee joint is a significant measure of its function, describing the forward or backward movement of the tibia in relation to the femur during motion (Liu et al., 2024). During knee flexion in normal movement, the tibia shifts anteriorly, and during extension, it moves posteriorly. Our study observed that the Contra-TKA group exhibited a reduced overall anterior translation during walking compared to the Contra-OA group, with a particularly notable decrease at the IC, OT, OI, TO and TV moment. The observed reduction in anterior tibial translation during knee flexion in the Contra-TKA group is likely due to the enhanced support from the operated knee joint following TKA. With this increased support, the non-operated knee joint is relieved of the need for excessive anterior movement of the tibia, which simplifies the walking motion and contributes to decreased pain. This indicates that the TKA procedure might not only improve the function of the operated knee but also positively affect the biomechanics and comfort of the non-operated knee, promoting overall better gait efficiency and reducing the load on the contralateral joint.

The scope of our study has several limitations that should be noted. Firstly, the study focused exclusively on patients who underwent unilateral TKA with a gap of at least 6 months between surgeries, excluding those with shorter intervals. This exclusion may have limited our understanding of the immediate and short-term effects of TKA on the contralateral knee. Future research will aim to address this by including patients with shorter intervals. Secondly, the study did not incorporate patient-reported outcomes, which are crucial for evaluating the subjective experience of pain relief and functional improvement following TKA. The inclusion of such data will enhance the depth of our analysis and provide a more patient-centered perspective. Thirdly, the study sample was restricted to end-stage osteoarthritis patients with varus deformity, excluding those with less severe OA, a healthy control group, and patients with valgus deformity. This limitation may have restricted the generalizability of our findings. Future studies will aim to include a more diverse patient population to broaden the applicability of our results and to better understand the impact of TKA across a wider range of conditions and patient experiences.

## 5 Conclusion

In summary, the study’s findings indicates that unilateral TKA provides kinematic effects on the untreated contralateral knee. This is demonstrated by notable differences in step length and in various rotation and translation gait parameters. The observed enhancements imply a constructive biomechanical alteration following TKA on the untreated knees. Consequently, this might offer a measure of support to end-stage OA patients, potentially affecting the progression of osteoarthritis and the overall gait pattern.

## Data Availability

The original contributions presented in the study are included in the article/supplementary material, further inquiries can be directed to the corresponding authors.
